# Efficient biosynthesis of (*R*)-mandelic acid from styrene oxide by an adaptive evolutionary *Gluconobacter oxydans* STA

**DOI:** 10.1186/s13068-023-02258-7

**Published:** 2023-01-13

**Authors:** Fei Liu, Junping Zhou, Mengkai Hu, Yan Chen, Jin Han, Xuewei Pan, Jiajia You, Meijuan Xu, Taowei Yang, Minglong Shao, Xian Zhang, Zhiming Rao

**Affiliations:** 1 Key Laboratory of Industrial Biotechnology of the Ministry of Education, Laboratory of Applied Microorganisms and Metabolic Engineering, School of Biotechnology, Jiangnan University, Wuxi, 214122 China; 2 School of Biotechnology, Zhejiang University of Technology, Hangzhou, 310014 China

**Keywords:** (*R*)-mandelic acid, *Gluconobacter oxydans*, Adaptive laboratory evolution, Promoters, Styrene oxide, Biotransformation

## Abstract

**Background:**

(*R*)-mandelic acid (*R*-MA) is a highly valuable hydroxyl acid in the pharmaceutical industry. However, biosynthesis of optically pure *R*-MA remains significant challenges, including the lack of suitable catalysts and high toxicity to host strains. Adaptive laboratory evolution (ALE) was a promising and powerful strategy to obtain specially evolved strains.

**Results:**

Herein, we report a new cell factory of the *Gluconobacter oxydans* to biocatalytic styrene oxide into *R*-MA by utilizing the *G. oxydans* endogenous efficiently incomplete oxidization and the epoxide hydrolase (SpEH) heterologous expressed in *G. oxydans*. With a new screened strong endogenous promoter P_*12780*_, the production of *R*-MA was improved to 10.26 g/L compared to 7.36 g/L of using P_*lac*_. As *R*-MA showed great inhibition for the reaction and toxicity to cell growth, adaptive laboratory evolution (ALE) strategy was introduced to improve the cellular *R*-MA tolerance. The adapted strain that can tolerate 6 g/L *R*-MA was isolated (named *G. oxydans* STA), while the wild-type strain cannot grow under this stress. The conversion rate was increased from 0.366 g/L/h of wild type to 0.703 g/L/h by the recombinant STA, and the final *R*-MA titer reached 14.06 g/L. Whole-genome sequencing revealed multiple gene-mutations in STA, in combination with transcriptome analysis under *R*-MA stress condition, we identified five critical genes that were associated with *R*-MA tolerance, among which AcrA overexpression could further improve *R*-MA titer to 15.70 g/L, the highest titer reported from bulk styrene oxide substrate.

**Conclusions:**

The microbial engineering with systematic combination of static regulation, ALE, and transcriptome analysis strategy provides valuable solutions for high-efficient chemical biosynthesis, and our evolved *G. oxydans* would be better to serve as a chassis cell for hydroxyl acid production.

**Supplementary Information:**

The online version contains supplementary material available at 10.1186/s13068-023-02258-7.

## Background

An important challenge in the bio-manufacturing of high-value natural and unnatural chemicals is to develop green, efficient, and promising synthetic routes from cheap and readily available substrates. Epoxides are primarily from easily obtainable petroleum-based by-products and have broad applications in preparing pharmaceutical and fine chemicals [1–3]. Recently, epoxides have attracted much attention for hydrolyzing chiral vicinal diols to further synthesize unnatural compounds [4]. Our previous work had provided a self-sufficient cascade to biocatalytic epoxides to produce 1,2-amino alcohols [5].

The chiral (*R*)-mandelic acid (*R*-MA), as a useful chiral building block for the synthesis of aromatic drugs, is of crucial importance in the chemical and pharmaceutical industry [6–8]. Optically pure *R*-MA is an important intermediate for preparing many chiral drugs, including antitumors, antiobesity agents, antibiotics [9, 10]. In addition, MA also can be used to synthesize chiroptical materials [11–13]. Nowadays, *R*-MA is mainly produced by chemical synthesis with many by-products. Conversely, biosynthesis is an attractive alternative way to produce *R*-MA, which is non-toxic and has mild reaction conditions with high selectivity. It is an attractive and desirable way to produce *R*-MA using cheap styrene oxide as a substrate. However, the bioproduction of this valuable compound is still faced many challenges, including the lack of suitable catalysts and host strains for efficient synthesis reaction.

Recently, metabolic engineering in *Saccharomyces cerevisiae* and *Escherichia coli* and synthetic biology of multi-enzyme artificial cascades were used to synthesize *R*-MA [14, 15]. It is worth mentioning that they successfully produced *R*-MA from simple and readily available chemicals with high selectivity, whereas the yields are still not high. Among the cascade catalytic pathway described above, we noticed the accumulation of *R*-1-phenyl-1,2-ethanediol (*R*-PEG), which is probably because of the low expression and insufficient activity of alditol oxidase used to convert *R*-PEG to *R*-MA. In addition, an alcohol dehydrogenase and a phenylacetaldehyde dehydrogenase were assembled to biocatalyze *S*-PEG to *S*-MA [16], but there are few aldehyde dehydrogenases for the oxidation to *R*-MA.

The gram-negative obligate aerobic bacteria, acetic acid bacterium *Gluconobacter oxydans*, is famous for its stereo-selective incomplete oxidate sugars, alcohols, and polyols to the corresponding ketones and acids. As a wildly used industrial bacteria, *G. oxydans* has been successfully used to produce vitamin C, dihydroxyacetone, gluconic acid, and miglitol [17, 18]. Furthermore, it was reported that *G. oxydans* also was applied in oxidating *R*-PEG to *R*-MA by resting cells [19–22]. The conversion of the overwhelming majority of substrates and release of products do not need to transport the cell membranes, because the reactions typically occur in the periplasm using membrane-bound dehydrogenases, considerably improving the biosynthesis efficiency [23–25]. Hence, we selected *G. oxydans* as a host cell to construct a catalytic system for producing *R*-MA from styrene oxide.

However, studies have demonstrated that *R*-MA is toxic to *G. oxydans* and strongly inhibits to the oxidation reaction [19]. It may be a promising way to accelerate the catalytic efficiency and production by improving the *R*-MA tolerance of *G. oxydans*. Adaptive laboratory evolution (ALE) as an accessible and powerful approach in microbial engineering has become much more popular to obtain specially evolved strains with increased ability to survive under extreme conditions, inhibited metabolites, and toxic substrates or products [26–31]. Furthermore, ALE also successfully selected thermotolerant strains with improved growth and ethanol production in *S. cerevisiae* [32]. Recently, a 420-day adaptive laboratory evolution of *G. oxydans* was applied, resulting in a highly improved conversion efficiency of non-glucose sugars to sugar acids [33]. All the cases indicated that ALE is a powerful and efficient strategy to push microbe breakthrough the limit to synthesize toxic compounds.

**Fig. 1:**
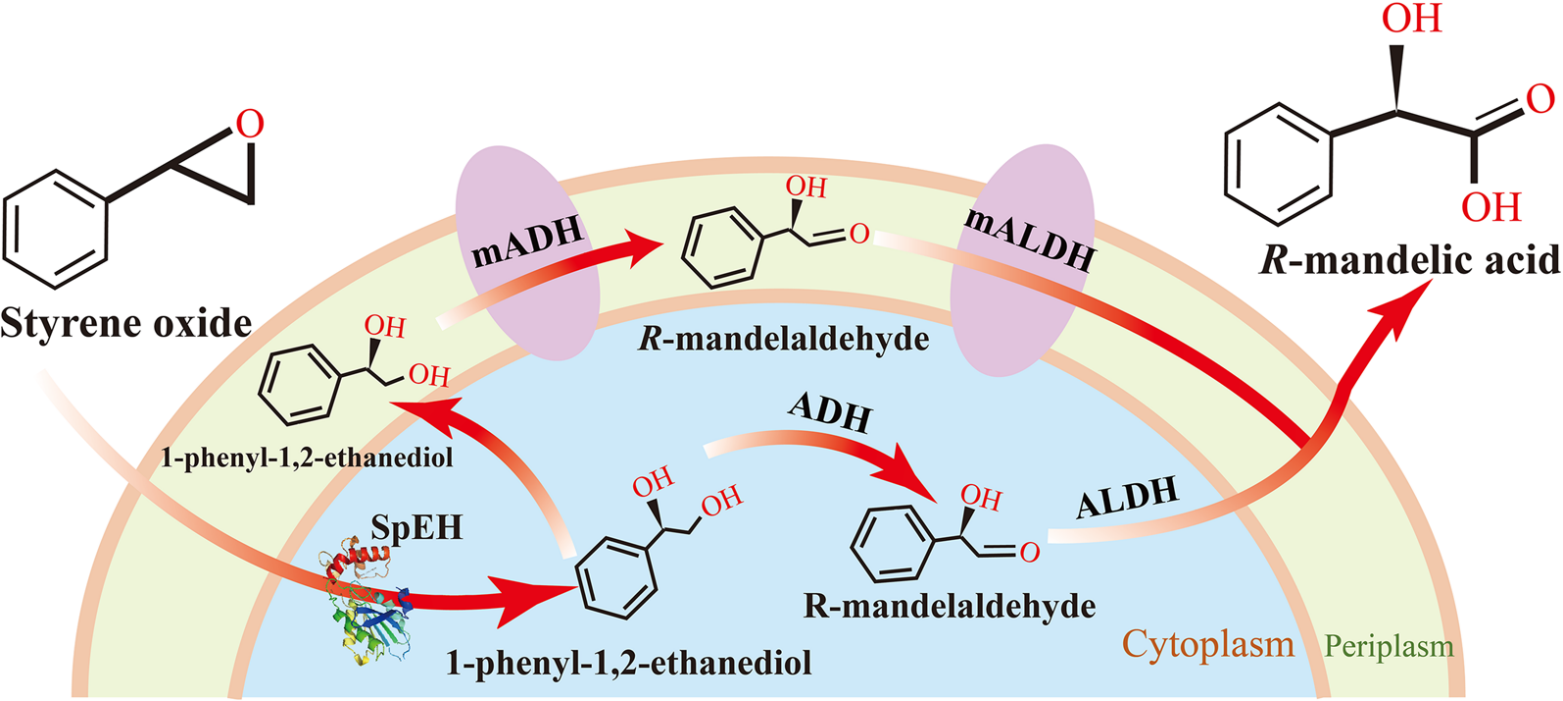
Scheme of bioproduction of *R*-MA from styrene oxide with recombinant *G. oxydans* 621H expressing epoxide hydrolase (SpEH) from *Sphingomonas *sp*.* HXN-200

In this study, we initially constructed an efficient catalytic system for producing *R*-MA from styrene oxide by heterologous expression of SpEH with a new screened endogenous strong promoter in *G. oxydans* (Fig. 1). Followed by the ALE strategy, an evolved *G. oxydans* named STA with enhanced *R*-MA tolerance ability was isolated and characterized. Then, we used transcriptome analysis of wild-type strain and STA to study their genetic mechanisms of the STA’s improved *R*-MA tolerance. Multiple vital genes involved in transcript levels changes were investigated, among which AcrA was confirmed to be essential for *R*-MA tolerance, STA-Δ*acrA* cannot even survive under a low concentration of *R*-MA. The evolved *G. oxydans* STA showed great potential for highly efficient *R*-MA production.

## Results and discussion

### Developing a biocatalytic cascade and screening new strong promoters for *R*-MA production in *G. oxydans*

To construct a biosynthesis cascade to product *R*-MA from styrene oxide, epoxide hydrolase (SpEH, GenBank ID: ANJ44372.1) from *Sphingomonas sp.* HXN-200 [5, 34] was chosen as the first step for hydrolysis of styrene oxide into *R*-PEG. In addition, *G. oxydans*, a famous non-pathogenic and safe microorganism, has shown strong incompletely oxidize capacity to produce alcoholic acid including *R*-MA. Thus recombinant *G. oxydans* with heterologous expressing SpEH was constructed for highly efficient *R*-MA production from bulk chemical styrene oxide.

In synthetic biology, promoters and ribosome binding site are critical in controlling protein expression and gene regulation [35–37]. As strong promoters are essential for enhancing the expression of specific genes, employing a suitable and strong promoter is very important for *spEH*. However, the alternative constitutive strong promoters applied in *G. oxydans* 621H are still constrained. Thus, we aimed to screen out and identify strong endogenous promoters from *G. oxydans* to optimize the expression of SpEH.

Nowadays, numerous biological promoter engineers have been developed to model and design promoter libraries or screen strong endogenous promoters directly from the genome [38]. A range of studies have clarified that it is an accessible strategy to mine strong constitutive promoters based on RNA sequencing data. 25 genes with constitutive strong promoters in *Methylotuvimicrobium buryatense* 5GB1 were identified, relying on whole genome and RNA-sequencing experimental data [39]. It was also reported that in *G. oxydans* WSH-003, a few strong promoters were obtained through RNA-sequencing data, and with the strongest promoter P_*2703*_ expressing SDH, the titer of 2-keto-l-gulonic acid increased distinctly [40].

**Fig. 2:**
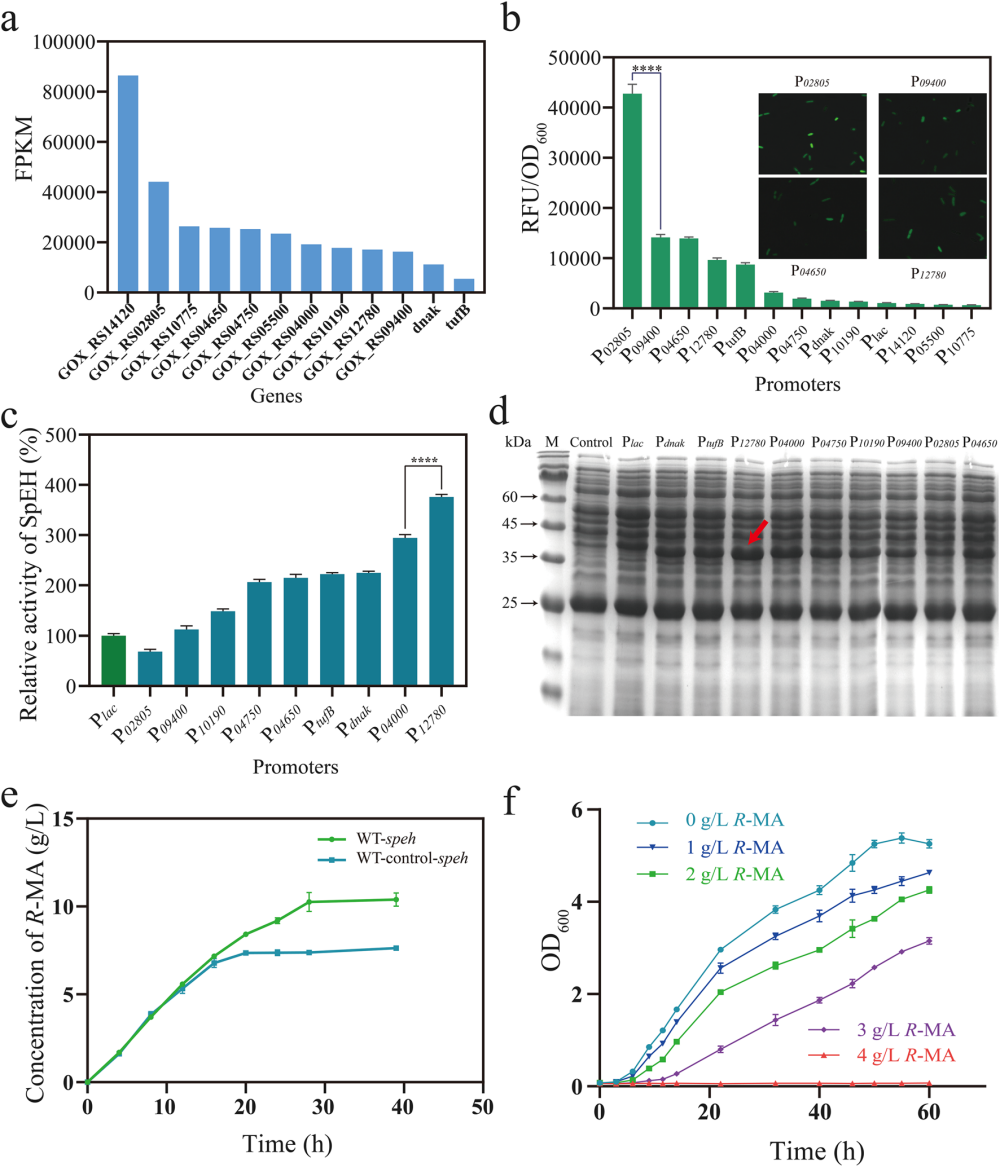
**a** FPKM value of 10 top strongly transcribed genes of *G. oxydans* 621H. **b** Strength of screened promoters evaluated with eGFP as reporter and the microscope pictures of P_*02805*_, P_*09400*_, P_*04650*_ and P_*12780*_. **c** Relative activity of SpEH. SpEH were expressed with screened and reported promoters. The enzyme activity of SpEH controlled by P_*lac*_ was set to 100% and its enzyme activity was 5.69 ± 0.26 U/mL. ****P < 0.0001. **d** SDS–PAGE of *SpEH* expression strains with the screened promoters. Lane M: protein marker; Lane 1: whole cell protein of *G. oxydans* 621H without overexpressing SpEH; Lanes 2–11: whole cell protein of recombinant *G. oxydans* strains with SpEH expressed by P_*lac*_, P_*dnak*_, P_*tufB*_, P_*12780*_, P_*04000*_, P_*04750*_, P_*10190*_, P_*09400*_, P_*02805*_, P_*04650*_. **e** Biotransformation time course of styrene oxide to *R*-MA of WT-*speh* and WT-control-*speh* cells. **f** Growth curves for *G. oxydans* 621H under 0, 1, 2, 3 and 4 g/L *R*-MA

In RNA-sequencing data, the promoters of genes with high fragments per kilobase million (FPKM) values may be potentially strong. First, we performed RNAseq analysis on *G. oxydans* 621H, relying on transcriptome results of *G. oxydans*, the 10 top FPKM values of genes were chosen (Fig. 2a, Additional file 1: Table S3). To evaluate the strength, all the 10 putative promoters (500-bp upstream fragments), as well as the reported strong promoters P_*dnak*_ and P_*tufB*_ from *G. oxydans* [41, 42] fused with e*gfp* gene (GenBank ID: AAK08507.1) were ligated to pBBR1MCS-2 and transferred into *G. oxydans*. By assaying the fluorescence intensity, seven strong promoters were identified compared to the control, including P_*02805*_, P_*09400*_, P_*04650*_, P_*12780*_, P_*04000*_, P_*04750*_, and P_*10190*_. Furthermore, the green fluorescence also was observed (Fig. 2b). Then, we heterologous expressed SpEH in *G. oxydans* 621H with these new promoters. The expression results of SpEH were displayed by SDS–PAGE and their crude enzyme activities (obtained from the same growth and cell density) also were detected (Fig. 2c, d). It can be clearly seen that P_*12780*_ had the highest expression and the activity was higher than P_*02805*_ and P_*04650*_ for 5.5 and 1.8 times, respectively. The results indicated that the fluorescent protein's intensity can only illustrate the strength of promoters to a certain extent and cannot accurately represent the expression level of a specific protein [43, 44]. As P_*12780*_ was the most suitable promoter for the expression of SpEH in *G. oxydans* (WT-*speh*), it was used for the transformation of styrene oxide into *R*-MA and the original promoter of pBB vector was used as control (WT-control-*speh*). Compared to 7.36 g/L *R*-MA of the control, WT-*speh* got better production of 10.26 g/L (Fig. 2e).

### ALE of *G. oxydans* and phenotypic characterization of the evolved strain

**Fig. 3:**
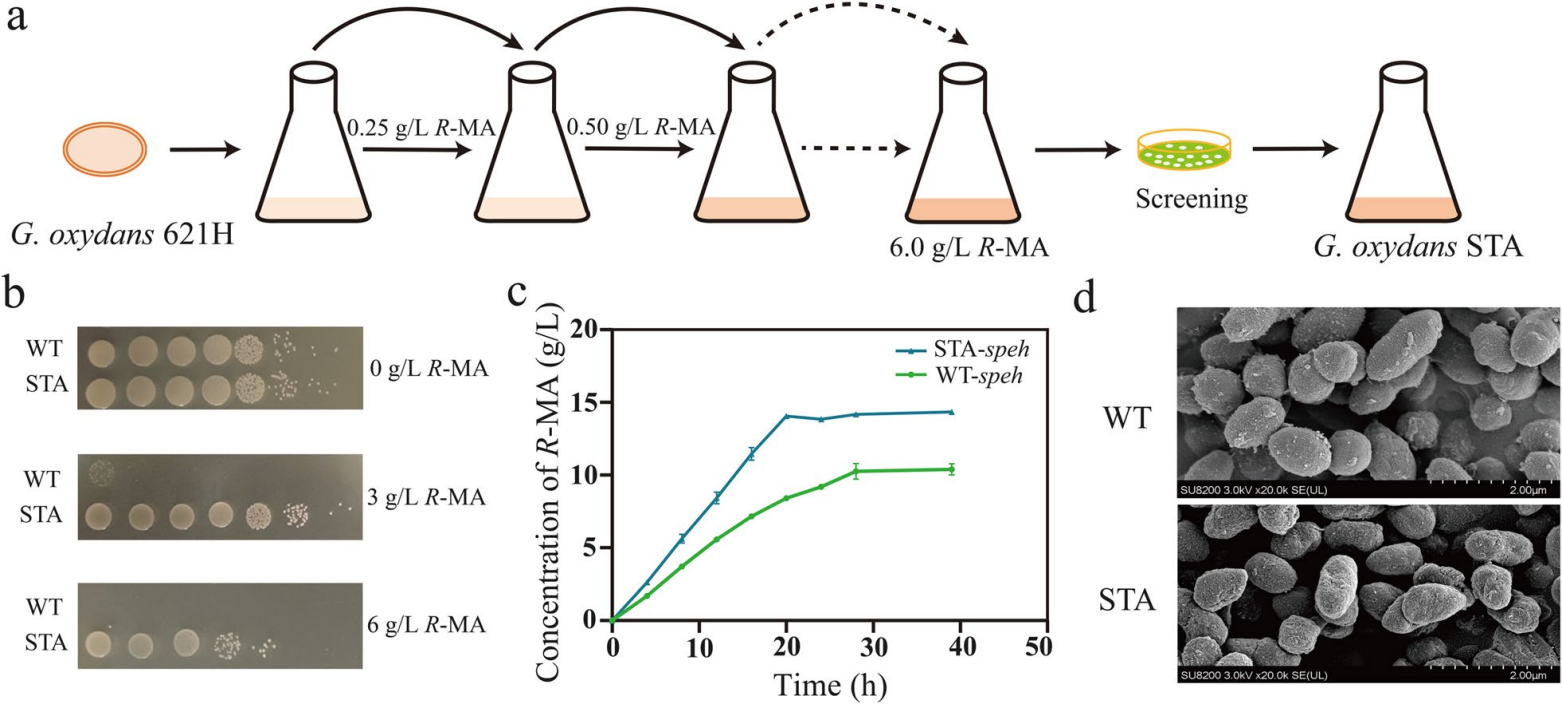
Adaptive laboratory evolution of *G. oxydans* to improve *R*-MA tolerance. **a** Schematic diagram of adaptive evolution. The original strain was *G. oxydans* 621H (WT). **b** Spot assays in *G. oxydans* WT and STA with 0 g/L, 3 g/L and 6 g/L *R*-MA. **c** Biotransformation time course of styrene oxide to *R*-MA of WT-*speh* and STA-*speh* cells. **d** Measurements of cell shape and length in wild-type *G. oxydans* and adapted strain STA

As it was reported that *R*-MA was toxic to *G. oxydans*, we then verified its survivability to *R*-MA. By analyzing the growth curve, the tolerance of *G. oxydans* 621H to different concentrations of *R*-MA was determined. The growth rate delayed obviously with 3 g/L *R*-MA and the cells cannot grow when cultured with 4 g/L *R*-MA (Fig. 2f). Apparently, *G. oxydans* was very sensitive to *R*-MA. Typically, the capacity to tolerate high-concentration product is a prospective characteristic for high production of target compounds [45]. To further improve the production of *R*-MA, the ALE strategy, an efficient approach to generating strains with desired phenotypes under selection pressure, was performed to enhance *R*-MA tolerance in *G. oxydans*. As shown above, the cell growth of *G. oxydans* was affected even with very low *R*-MA concentration. Thus, we determined the starting point for ALE in a much lower concentration of 0.25 g/L *R*-MA. The wild-type *G. oxydans* were cultured for 24 h, then transferred into a fresh medium with 0.25 g/L *R*-MA and 5% inoculation volume. After incubation of 24–48 h, at log phase, these bacteria were transferred into a fresh medium containing 0.5 g/L *R*-MA. Repeat these transfers every time with 0.25 g/L *R*-MA increased (Fig. 3a). As the increase of the concentration, adapted *G. oxydans* strains need much more time to grow to log phase, at that time, we cultured the strains in the same *R*-MA concentration more times to improve the cell stability. The final concentration of *R*-MA was 6 g/L; after screening, the evolved strain named STA was isolated. To assess the genetic stability of STA, we transferred log-phase cells into a fresh medium without *R*-MA every 24 h for 30 days. At the last time of transfer, 6 g/L *R*-MA was contained in the culture medium and STA can grow as before which indicates that the *R*-MA tolerance of STA can be stably inherited (data not shown).

To probe the phenotypic characterization, the survival assay was used between *G. oxydans* WT and STA. STA can remain viable in the plate with 6 g/L *R*-MA but the wild-type strain had no capacity to survive (Fig. 3b). As P_*12780*_ was the most suitable promoter for expressing SpEH, we then transformed the recombinant plasmid pBB-P_*12780*_-*speh* into STA (STA-*speh*). Recombinant strains were subsequently used to transform styrene oxide into *R*-MA (Fig. 3c). It can be clearly seen that STA-*speh* showed better catalytic ability, which can produce14.06 g/L *R*-MA but WT-*speh* 10.26 g/L. Most importantly, conversion rate increased from 0.366 to 0.703 g/L/h. In addition, the expression levels of SpEH in these two strains were comparable and their enzymatic activities were almost the same (Additional file 1: Fig. S1).

Since SpEH was expressed intracellular, styrene oxide needs to be transferred to the intracellular to be hydrolyzed into *R*-PEG, while *R*-PEG also needs re-transmembrane to periplasmic space to be further oxidized by membrane-bound alcohol and aldehyde dehydrogenase to *R*-MA. Hydrophobic substrate styrene oxide conversion to *R*-MA requires two transmembrane operations in this biotransformation cascade. Thus, it would be very important to study cell characteristics for the STA strain to understand how the catalytic efficiency was improved after ALE strategy. Previous studies have certified that the membrane properties such as permeability, hydrophobicity, and integrity of evolved strains were found to be contributing to the increasing tolerance [46–48]. Compared to the wild-type strain, the evolved carboxylic acids tolerant *E. coli* strain with increased membrane rigidity and decreased fluidity was proved to have a fivefold increase in titer [49]. Initially, their morphologies were examined by scanning electron microscopy. In control, STA as well as the wild-type strain was both grown in fresh medium, their cells were of short rod shape, whereas STA cells were much less and shorter than the parent one (Fig. 3d). In all probability, the membrane properties of STA have changed compared to the wild-type strain.

**Fig. 4:**
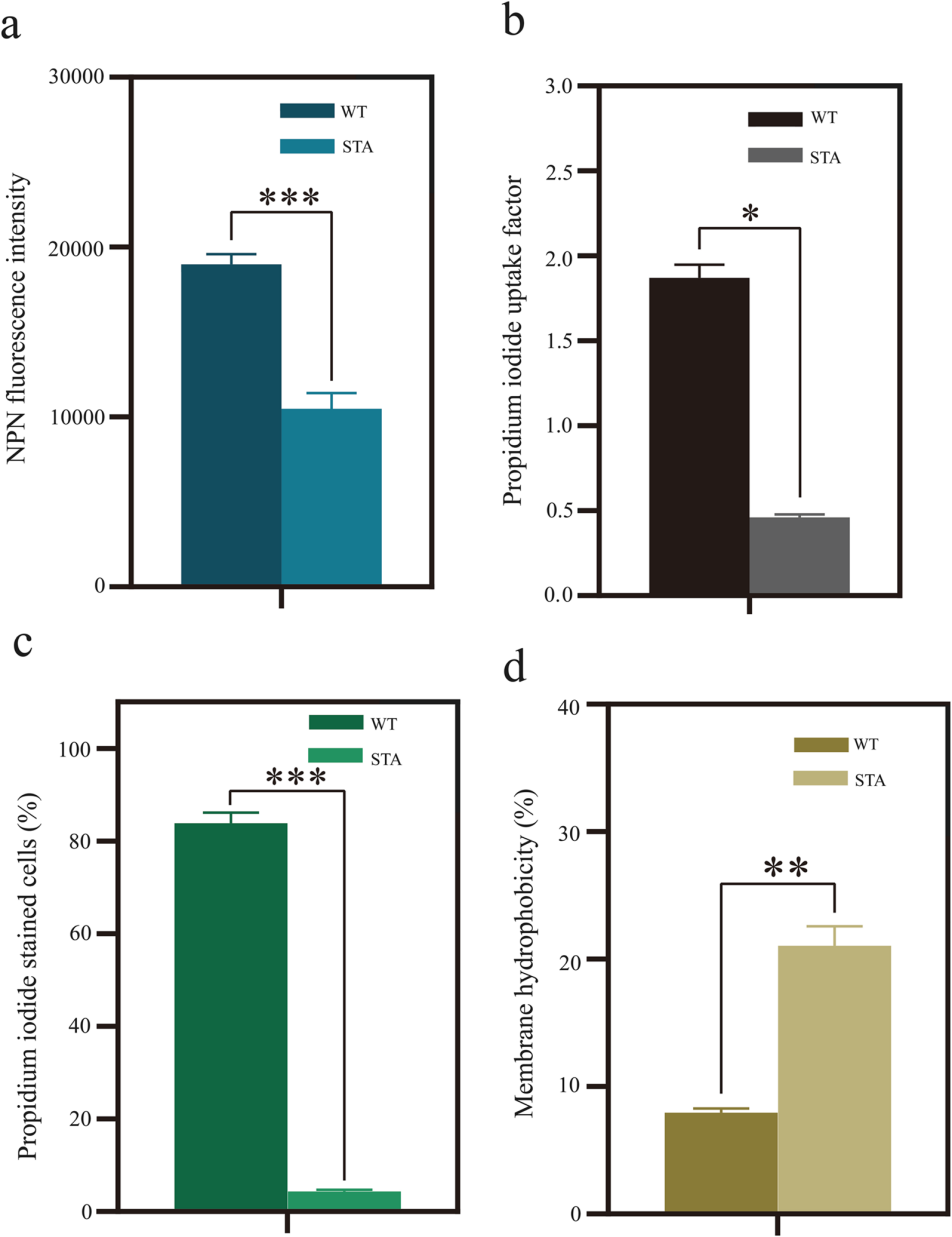
Membrane integrity, permeability and hydrophobicity analysis of *G. oxydans* WT and the adapted strain STA. **a** NPN fluorescence intensity analyses of outer membrane permeability in WT and STA. **b** Inner membrane permeability change of propidium iodide (PI) uptake factor in WT and STA. **c** Flow cytometry analyses of membrane integrity in WT and STA. Cells were stained with PI. **d** Membrane hydrophobicity was changed in WT and STA. *P < 0.1. **P < 0.01. ***P < 0.001

We first investigated the inner and outer membrane permeability of the parent strain and STA assessed by hydrophilic probe propidium iodide (PI) and hydrophobic probe N-phenyl-1-naphthylamine (NPN) uptake analysis. As shown in Fig. 4a, b, STA exhibited a decrease in the fluorescence intensity of NPN with almost 50% and its PI absorption factor was only one-quarter of the wild-type strain, indicating that STA had a poor permeability membrane. PI emit a strong fluorescence signal when they transport into cells and bind to nucleic acids. As a result, PI also can serve as a probe to investigate membrane integrity by flow cytometer. The ratio of PI-stained cells of the wild-type strain was 84.2%, much higher than STA (Fig. 4c), which signified that STA had increased the membrane integrity. Interestingly, improved membrane integrity was shown to enhance the fatty acid tolerance and the final titer in *E. coli* [50].

Membrane hydrophobicity was another critical characteristic for cell viability and bioconversion activity. The STA strain had a percentage of adhesion of 21.0% relative to the control of 7.9%, which represented that the adaptive evolution of *G. oxydans* had significantly improved the membrane hydrophobicity (Fig. 4d). High cell surface hydrophobicity helped to against multi environmental stressors and reduce the distance of cell to hydrophobic substrate for the enhancement of transport rate [51, 52]. In summary, STA had worse membrane permeability and better membrane integrity, most importantly, the membrane hydrophobicity had increased. These membrane characteristics changes provide new insights into the understanding of *R*-MA tolerance and increasement of catalytic efficiency by STA.

### Global transcriptome analysis and whole-genome sequencing of *G*. *oxydans* WT and STA

**Fig. 5:**
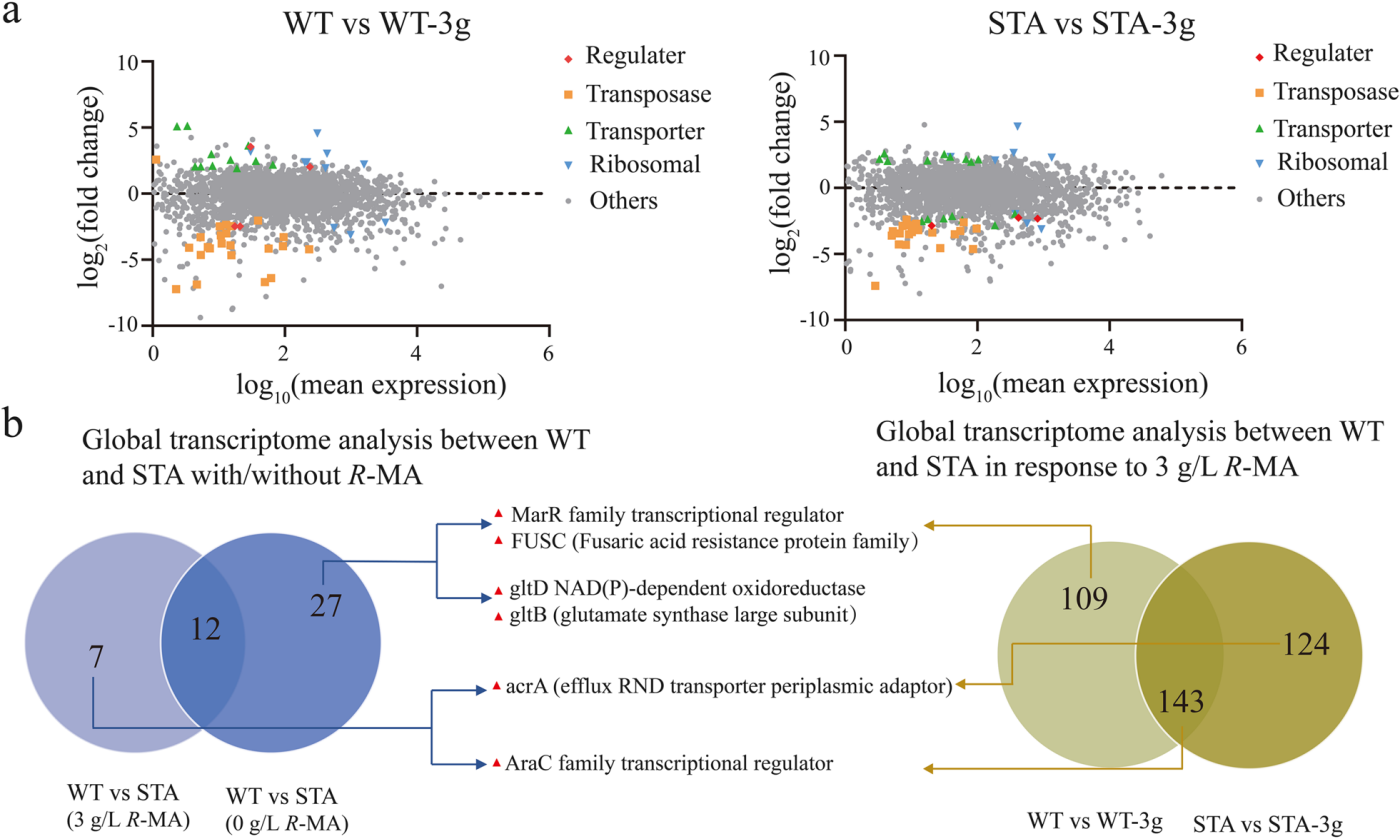
Transcriptome analysis of *G. oxydans* WT and STA. **a** Left side displays the gene expression differences of *G. oxydans* WT under normal and 3 g/L *R*-MA condition. The right side displays the gene expression differences of the adapted strain STA under normal and 3 g/L *R*-MA condition. *X-axis* represents the logarithmic conversion value of gene expression in wild type strain (left side) or adapted strain (right side). *Y-axis* represents the log_2_-transformed value of expression change folds. Classification of genes with different functions are represented by different color shapes as indicating. Others representative genes are not part of the indicated classification. **b** Venn diagrams showing the regulated gene numbers between *G. oxydans* WT and STA under 0 g/L *R*-MA and 3 g/L *R*-MA conditions (left side) and the regulated gene numbers between *G. oxydans* WT and STA in response to 3 g/L *R*-MA (right side). Six common regulated genes were depicted in the middle

The differentially regulated genes in transcriptional levels were crucial factors for analyzing the cellular processes [53, 54]. To further explore the molecular mechanisms that accompanied *R*-MA tolerance of STA, transcriptome analysis was conducted to compare global gene expression in wild-type *G. oxydans* and STA under 0 g/L or 3 g/L *R*-MA condition. Comparative transcriptome data of wild-type *G. oxydans* with/without *R*-MA showed that the expression levels of 60 genes were significantly upregulated, while 192 genes were significantly downregulated at least twofold. By contrast, 58 genes upregulated and 209 genes downregulated at least twofold in STA (Fig. 5a, b). Only 143 commonly regulated genes are involved in wild-type *G. oxydans* and STA for the response to *R*-MA, which indicated that there must be some important genes regulated in STA relative to the tolerance of high *R*-MA concentration.

The regulated genes in STA relative to those in WT were analyzed under normal and 3 g/L *R*-MA treatment conditions. As shown in Fig. 5b, common regulated genes were identified in *G. oxydans* WT and STA. Among these regulated genes, the multiple antibiotic resistance regulator family (MarR) gene and fusaric acid resistance protein family (FUSC) gene were upregulated by 7.57-fold and 6.30-fold in STA compared to the wild-type strain in normal condition. Similarly, these two genes also were upregulated when the wild-type strain was cultured with 3 g/L *R*-MA condition. Notably, MarR family transcriptional regulator is known for regulating diverse cellular responses and physiological processes [55]. Another two genes, encoding AcrA (efflux RND transporter periplasmic adaptor) and AraC family transcriptional regulator, were up-regulated in STA under 3 g/L *R*-MA condition. In wild-type strain, AraC regulator also was upregulated when facing 3 g/L *R*-MA, while AcrA was not regulated significantly. We also observed GltD (NAD(P)-dependent oxidoreductase) and GltB (glutamate synthase large subunit) genes were upregulated in STA compared to WT without *R*-MA, on the contrary, they all downregulated when STA was cultured with 3 g/L *R*-MA.

To identify genetic mutations contributing to the improved tolerance of *R*-MA, whole-genome sequencing was performed on the evolved strain STA, relative to the published sequence for wild-type *G. oxydans* 621H. STA carrying a large number of mutations and all the mutations including single nucleotide variations (SNVs) and nucleotides insertion-deletion (InDel) were listed in Additional file 2: Table S4-1. Among the mutation genes, there were two nonsynonymous mutations in the coding regions of GltB and MarR family transcriptional regulator (their mutations were further identified by gene sequencing and listed in Additional file 2: Table S4-2), which had also been reported to be related to cellular physiological processes, these processes showed response to the extreme environment or associating with the stress or antibiotics resistance. Most importantly, these two genes also were upregulated significantly in response to *R*-MA condition. MarR family transcriptional regulator has been shown to regulate diverse cellular processes, including conferring resistance to antibiotics, organic solvents, and virulence [56]. The glutamate synthase GltB was proved to be involved in the biofilm formation as well as the oxidative stress tolerance in *Listeria monocytogenes* [57].

### Key genes that contribute to *R*-MA tolerance of *G*. *oxydans* STA

**Fig. 6:**
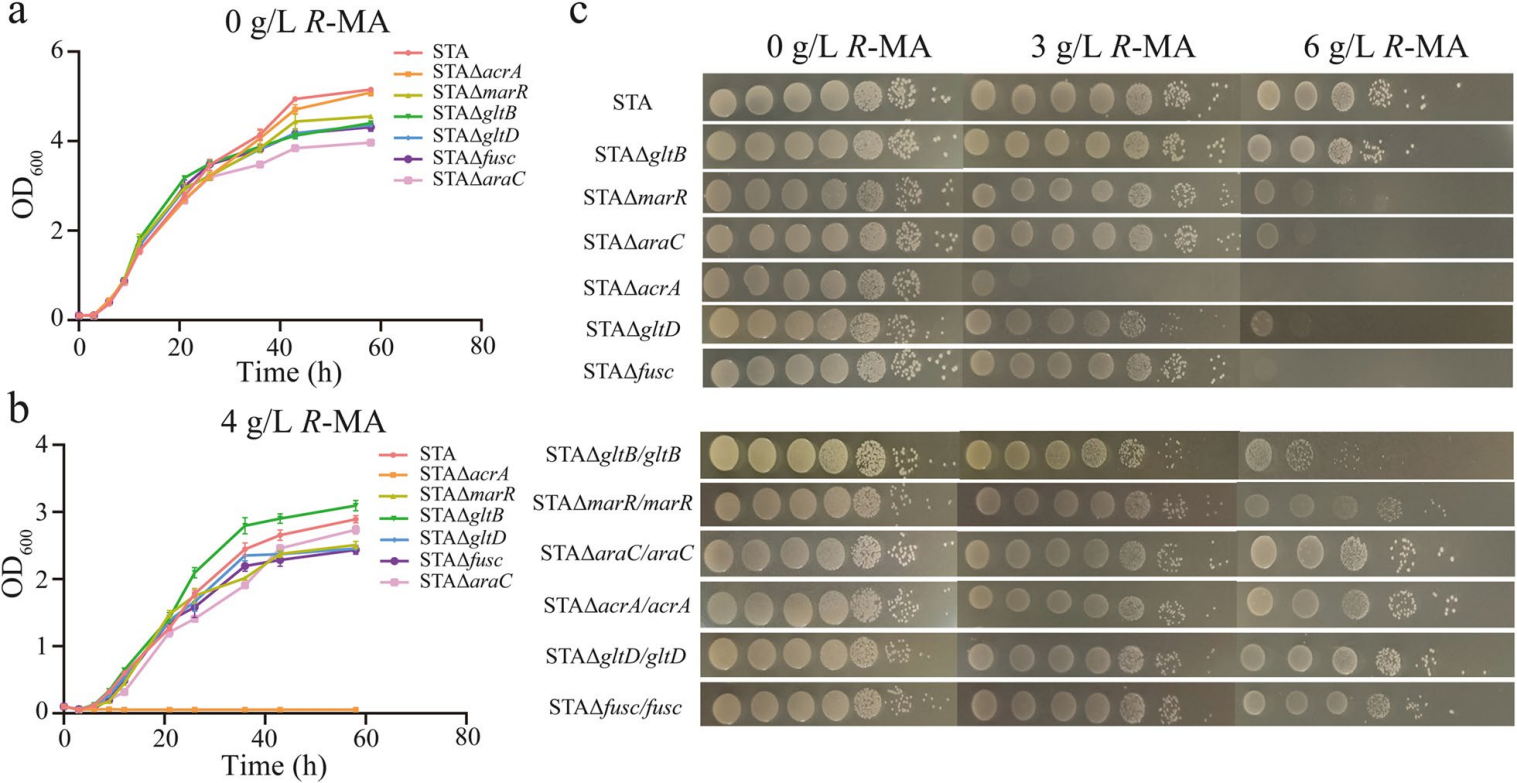
Growth in *G. oxydans* STA of different gene deletions to test their contribution for *R*-MA tolerance. **a** Growth curves of *G. oxydans* STA and its gene-deletion strains under normal condition. **b** Growth curves of *G. oxydans* STA and its gene-deletion strains in the presence of 4 g/L *R*-MA. **c** *G. oxydans* STA and its gene-deletion strains were spotted on solid medium under 0 g/L, 3 g/L and 6 g/L *R*-MA condition

To study the contribution of the genes mentioned above, single-knockout strains of *marR*, *fusc*, *gltD*, *gltB*, *acrA* and *araC* were constructed in STA. The cell growth was determined in normal medium. There are no significant growth differences between STA and STAΔ*acrA*, while deletion of other genes was slightly detrimental to cell growth (Fig. 6a). The growth curves under 4 g/L *R*-MA stress were investigated afterward. STAΔ*acrA* have no capacity to survive just like wild-type *G. oxydans*. While all others had a delayed log phase and declined final cell density compared with the control (Fig. 6b). Knockout GltB increased the growth ability of STA under 4 g/L *R*-MA condition. To directly display the impact of these genes, the single-knockout strains were spotted and grown on culture plates with 0, 3 and 6 g/L *R*-MA. Delete *marR*, *fusc*, *gltD*, *acrA* and *araC* caused significant growth defects in the presence of 6 g/L *R*-MA compared to STA. Notably, different with other strains can grow normally at 3 g/L *R*-MA stress condition, the growing vitality of STAΔ*acrA* had been greatly inhibited. We overexpressed the six genes in its corresponding knockout strains for further assessing their functions. As shown in Fig. 6c, five strains recovered the tole-rant phenotype, on the contrary, overexpress GltB caused a growth defect in 6 g/L *R*-MA stress. Clearly, the ability of STA to tolerant high-concentration *R*-MA required the interaction of multiple genes. Furthermore, as STA had changed the membrane properties compared to wild-type strain, we also explored the membrane properties of STA strains. As shown in Additional file 1: Fig. S2, compared to STA, STAΔ*gltB* had better membrane permeability and STAΔ*gltD* had worse membrane permeability. The membrane integrity and hydrophobicity of the strains was nearly the same.

**Fig. 7:**
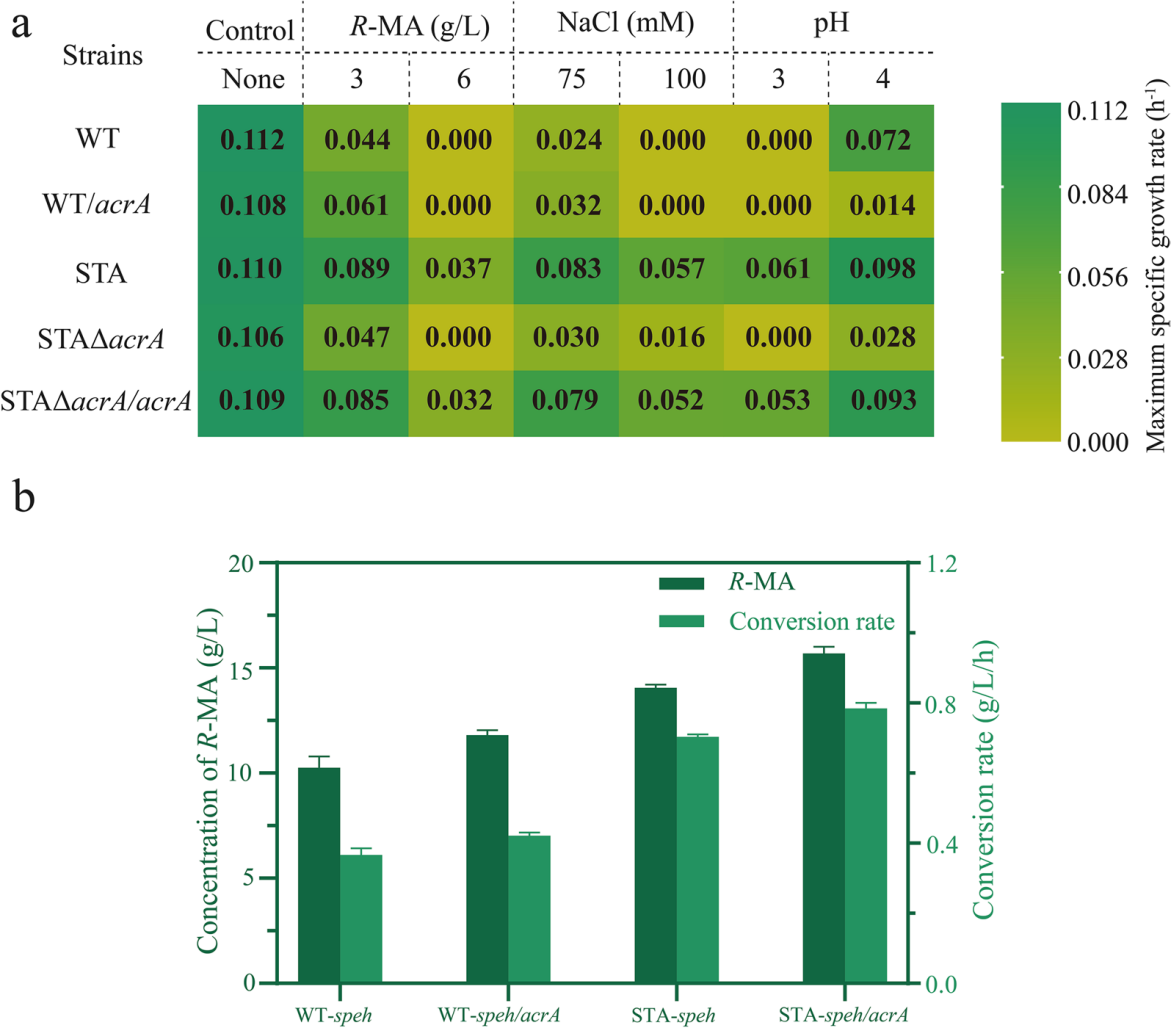
Contribution of AcrA for *G. oxydans* WT and *G. oxydans* STA in cell growth under different environmental stress and final *R*-MA production. **a** Growth characterizations of WT, STA and AcrA associated strains are represented by the maximum specific growth rates (h^−1^). Right bar indicates the color-scale of the growth rate. **b** Transformation of styrene oxide into *R*-MA by WT, STA and AcrA associated strains with SpEH or SpEH co-expressing with AcrA

MarR and AraC as transcription regulators both were proved to regulate the expression of diverse genes related to stress and virulence response [58, 59]. AraC family regulator YdeO enhanced the acid and the multi-drug resistance in *E. coli* [60]. Another key gene was *gltD*, which altered the critical biofilm properties for environmental adaptation [61]. We also noticed that only deletion of AcrA led to significant growth defect with 3 g/L *R*-MA, which indicates that AcrA may play the most vital role in the tolerance of STA to *R*-MA. Consequently, we decided to focus on AcrA for further evaluation. AcrA belongs to RND efflux pumps, which have an important contribution to antibiotic resistance and microbial environmental adaptability in bacteria [62, 63]. Then, further analysis was performed to test whether AcrA had associated with other phenotypes. Surprisingly, the evolved strain STA showed higher growth rate than the wild-type *G. oxydans* under osmotic and low-pH stress (Fig. 7a). The AcrA knockout strain of STA completely lost the ability to survive under pH 3 condition and the osmotic tolerance also greatly reduced with 75 mM NaCl. To further verify the functions, it also was overexpressed in wild-type *G. oxydans*. Interestingly, *G. oxydans*/*acrA* exhibited improved tolerances in these stress conditions. In addition, all strains had the highest growth rate when none extreme factor was present, indicating that the normal growth of *G. oxydans* does not depend on AcrA.

### Validation the role of AcrA in *R*-MA production

AcrA, an RND efflux transporter, was reported to be a multidrug transporter for extrusion of a broad range of compounds in gram-negative bacteria. It also supports bacterial adaptation in the absence of different niches [64, 65]. Through inhibited RND pump, the drug susceptibility of *E. coli* was enhanced [66]. It also reported that an RND transporter upregulated in ALE-derived *Pseudomonas putida* for toluene tolerance [67]. In this study, when *G. oxydans* STA suffered with 3 g/L *R*-MA, the transcriptional levels of *acrA* were upregulated by 2.2-fold. In addition, compared to wild-type strain in 3 g/L *R*-MA condition, *acrA* was upregulated by 3.9-fold. Nevertheless, it was only 1.3-fold upregulation in the case of normal condition as STA compared to WT. Considering the critical function of AcrA in STA when facing environmental pressure, it could be practical to further improve the *R*-MA production by enhancing AcrA expression. Then, we coexpressed AcrA and SpEH in *G. oxydans* strains, and not only the final production of *R*-MA increased, the conversion rate also was improved. Most importantly, the *R*-MA production was further improved to 15.70 g/L by the evolved strain (Fig. 7b), which was the highest obtained from styrene oxide reported thus far.

## Conclusions

In this study, *G. oxydans* was first used as a host cell for expressing SpEH to produce *R*-MA from bulk industrial chemical styrene oxide. After improving the *R*-MA tolerance of *G. oxydans* 621H by adaptive laboratory evolution together with the screened new strong promoter P_*12780*_ for enhancing SpEH expression, the production increased by 1.92-fold (from 7.36 to 14.06 g/L) and the conversion rate greatly improved from 0.366 to 0.703 g/L/h. We also described the membrane phenotype changes of the adapted *G. oxydans* STA, including better membrane integrity and increased membrane hydrophobicity, which may directly relate to the improved catalytic efficiency of organic substrate styrene oxide. Genomic sequencing and transcriptome analysis revealed that multiple key genes involved in enhancing *R*-MA tolerance. Finally, the *R*-MA production was further improved by an engineered *G. oxydans* STA. This study indicated that *G. oxydans* would be a promising host for the production of α-hydroxy acids from epoxides and its excellent ability to stereo-selective incomplete oxidation idols to the corresponding acids could be used to the maximum extent. In addition, ALE strategy combing with the comparative omics studies would be a valuable tool for enhancing chassis cell characteristics and guiding further metabolic engineering directions for high-value chemical production.

## Materials and methods

### Strains, media, and culture conditions

The *G. oxydans* strains used in this study are listed in Additional file 1: Table S1. *G. oxydans* cells were grown in the medium containing 8% sorbitol, 2% yeast extract, 0.1% KH_2_PO_4_, 0.05% MgSO_4_, 0.01% glutamine and incubated at 30 °C with shaking at 200 rpm. *E. coli* JM109 as host cell was used for plasmid construction and amplification and routinely grown at 37 °C in LB medium (0.5% yeast extract, 1% tryptone, 1% NaCl). 50 µg/mL kanamycin and 90 µg/mL gentamicin were used for selecting recombinant plasmids.

### Construction of recombinant strains

The e*gfp* and *speh* genes kept in our lab were amplified by PCR. All the screened potential promoters and the reported P_*tufB*_ and P_*dnak*_ were amplified by PCR with *G. oxydans* 621H genome as the temple. The sequences of primers used for engineering recombinant plasmids are listed in Additional file 1: Table S2. The amplified promoter fragments were ligated with the corresponding e*gfp* and *speh* genes, and inserted into the *Hind* III and *Xho* I site of pBBR1MCS-2. All the ligated productions were transformed into *E. coli* JM109 and followed by DNA sequencing to confirm the ligation results. Then, the engineered vectors were transformed into *G. oxydans*. The endogenous genes *marR*, *fusc*, *gltD*, *gltB*, *acrA*, and *araC* in *G. oxydans* STA were deleted by homologous recombination. We amplified their upstream and downstream homology arm fragments from genomic DNA of *G. oxydans* STA and ligated them with the marker gene of KAN amplified from pBBR1MCS-2. These fusion fragments were directedly introduced into *G. oxydans* STA, following which kan-resistant transformants were selected. Their genes were amplified and inserted into pBBR1MCS-5 using Gm antibiotics to select recombinant plasmids and then transformed into the corresponding single-knockout strains.

### Adaptive laboratory evolution experiments

A *G. oxydans* 621H colony was inoculated into a 10 mL medium to culture about 24 h to the logarithmic phase. Then, transferred into 50 mL fresh medium containing 0, 1, 2, 3 and 4 g/L *R*-MA, respectively, monitored their growth by measuring the cell density at OD_600_. For the adaptive evolution experiments, the strategy of inoculating *G. oxydans* with gradually increasing the concentration of *R*-MA, and the initial concentration was 0.25 g/L. After cultivating to the log phase, the cultivation was transferred into the fresh medium with an increasing concentration of *R*-MA by 0.25 g/L each time at 5% inoculation volume. After a long term of adaption, the adaptive strains can survive in the medium containing 6 g/L *R*-MA, and we screened the single colony by plating on the plates with 6 g/L *R*-MA and named *G. oxydans* STA. To verify the genetic stability of *G. oxydans* STA, we cultured the single colonies of wild type *G. oxydans* and STA to log phase and then transferred them into fresh medium without *R*-MA every 24 h. After inoculating for 30 days, the cultivations were into fresh medium containing 6 g/L *R*-MA to observe their growth.

### Spot assays and cell growth assays

*G. oxydans* was cultivated to logarithmic phase then diluted to an absorbance at 600 (OD_600_) of 2.0 in fresh medium, then aliquots of tenfold serial dilutions (4 µL) were spotted onto sorbitol plates containing no *R*-MA or indicated concentrations of *R*-MA. The cells were inculcated for 3 days in 30 °C.

*G. oxydans* strains were inoculated into 10 mL medium to culture about 24 h to the logarithmic phase and then transferred into 50 mL fresh medium containing 0, 1, 2, 3 and 4 g/L *R*-MA, respectively. We recorded the OD_600_ values at regular time intervals.

### Transcriptome analysis and whole-genome sequencing

After the ALE assay, the endpoint colony of STA was selected for transcriptome analysis. *G. oxydans* WT and *G. oxydans* STA cells were cultured to a same OD_600_ of 2.3, then the cells were collected, rapidly frozen by liquid nitrogen. RNA extraction and transcriptome sequencing were performed by GENEWIZ Biotech Co., Ltd. (Suzhou, China). All differentially expressed genes were determined GENEWIZ (Suzhou, China). All differentially expressed genes were determined by q-value ≤ 0.05 and |log2ratio| of ≥ 1 (Additional file 2: Table S4). The genome of *G. oxydans* 621H (NC_006677.1) was used as the reference [68].

Then, a colony of STA was selected for whole-genome sequencing. 100 ng genomic DNA was randomly fragmented by sonication (Covaris S220) to a size of less than 500 bp. Then, end repairing, A-tailing and adding adaptors were treated to these fragments using End Prep Enzyme Mix. After amplification and purification, the products were validated using an Agilent 2100 Bioanalyzer. A PacBio sequencing library was constructed and then sequenced using the Sequel II sequencing platform. Based on de novo analysis, mutations of insertions, deletions, and single nucleotide mutations were identified with the wild-type *G. oxydans* 621H genome sequence as reference.

### Fluorescence intensity assay and enzyme activity assays of SpEH

*G. oxydans* strains carrying the eGFP express plasmids of different promoters were cultivated in the logarithmic phase, collected and washed with 0.2 M PB buffer (pH 7.0) and resuspended in PB buffer at an appropriate concentration. The cell fluorescence was measured using Gen5 Data Analysis Software (BioTek, USA) at an excitation of 488 nm and an emission of 509 nm upon measuring 96-well plates. The normalized activity of eGFP was defined as the ratio of the fluorescence unit (RFUs) divided by the absorbance at 600 nm.

*G. oxydans* strains were cultivated to a same cell density in log phase, harvest, washed and resuspended with phosphate buffer (PB, 200 mM, pH 7.0) to be sonicated under the ice bath to obtain the crude enzyme solution and prepared a 0.2 M ethylene oxide substrate solution in methanol. 850 µL PB buffer mixed with 100 µL crude enzymes were placed at 35 °C for 5 min. The reaction started by adding 50 µL substrate solution and incubated for 10 min. The reaction was terminated by being treated in a boiling water bath for 10 min. The enzymic activity of SpEH was analyzed using high-performance liquid chromatography (HPLC). One unit of SpEH activity was defined as the amount of enzyme needed to hydrolyze ethylene oxide to produce 1 μmol 1-phenyl-1,2-ethanediol (PEG) per minute.

### Biotransformation procedure to convert styrene oxide to *R*-MA with resting cells of *G*. *oxydans* strains

The SpEH expression strains of *G. oxydans* were cultured in 10 mL medium containing 50 µg/mL kanamycin and then transformed into 100 mL medium for 30 h to reach the logarithmic phase. The cells harvested by centrifuge (8000 rpm, 4 °C, 5 min) and washed twice, then resuspended in PB buffer. The fresh cell density of the biotransformation mixture is 25 of OD600 in a 10 mL system, and the initial concentration of styrene oxide is 4 g/L, then added 4 g/L every 4 h until it reached 20 g/L. The reaction was carried out in a 50 mL shaker at 30 °C, 200 rpm, and 100 µL aliquots of the mixture were taken out every 4 h before adding substrates. Then, centrifuged at 10,000 rpm for 1 min, 50 µL of supernatant was diluted with 950 µL ultrapure water and then filtered by 0.22 µm filter for HPLC analysis. R-MA was analyzed by HPLC equipped with an Aminex HPX-87H analysis column (Bio-Rad, 300 × 7.8 mm) and UV–Vis detector at 210 nm using 5 mM H_2_SO_4_ as eluent [69].

### Membrane characteristics analysis

*G. oxydans* wild-type and *G. oxydans* STA cells were cultured to the same OD_600_ in log phase, then centrifuged and washed with PB buffer, and diluted to OD_600_ of 0.5. Propidium iodide (PI) was applied to analyze inner membrane permeability [70]. Taking 2 mL of dilutions into centrifuge tubes, 4 µL PI (1 g/L) were added and incubated in the dark for 5 min, harvested and washed twice. The controls were the same treatment using PB buffer replaced PI. The fluorescence intensity was measured under the excitation wavelength of 536 and emission wavelength of 617 using a fluorescence spectrophotometer. The inner membrane permeability was indicated by the absorption factor of PI and analyzed by the following formula: PI absorption factor = [F(PB + cells + PI)-F(PB + cells)]/[F(PB + PI)-F(PB)]. *N*-Phenyl-α-naphthylamine (NPN) was used to analyze the outer membrane permeability [71]. 200 µL sample dilutions were mixed with 2 µL NPN (10 mM), then the fluorescence intensity was detected by fluorescence spectrophotometer (excitation at 350 nm and emission at 420 nm).

The membrane integrity of *G. oxydans* strains were also analyzed using PI as the fluorescence dye, and the preparation of samples was the same as described above [72]. 500 µL dilutions with 3 µL PI were incubated in dark condition for 5 min, then measured by FACSCalibur flow cytometer with a rate of 500 to 800 cells/s and detected more than 20,000 cells. CellQuest software was used to collected and analyzed the results.

Membrane hydrophobicity of *G. oxydans* wild-type and *G. oxydans* STA cells were monitored by measuring the microbial adhesion to hydrocarbons (MATH) [46, 73]. After being collected and washed twice, the cells were suspended in PB buffer with a certain optical density at 550 nm (A0). 2.4 mL cell suspension together with 0.8 mL dodecane were vortexed at 1500 rpm for 10 min, then held for another 10 min to allow phase separation. Removed the organic layer and measured the absorbance (OD550) of the aqueous phase (A1). We used the following formula to calculate the percentage of cells into dodecane: (adhesion, %) = (A0–A1)/A0*100%. The adhesion was used as an indicator of the membrane hydrophobicity [74, 75].

## Supplementary Information

**Additional file 1: Fig. S1** (a) SDS–PAGE of *SpEH* expression strains. Lane M: protein marker; Lane 1: whole cell protein of wild-type *G. oxydans* without overexpressing SpEH; Lane 2: whole cell protein of WT-*SpEH*; Lane 3: whole cell protein of *G. oxydans* STA without overexpressing SpEH; Lane 4: whole cell protein of STA-*SpEH*. (b) Enzyme activity of crude SpEH of WT-*SpEH* and STA-*SpEH*. **Fig. S2.** Membrane permeability and hydrophobicity analysis of *G. oxydans* STA strains. (a) NPN fluorescence intensity analyses of outer membrane permeability in STA stains. (b) Inner membrane permeability change of propidium iodide (PI) uptake factor in STA stains. (c) Membrane hydrophobicity was changed STA stains. **P < 0.01. ***P < 0.001. **Table S1.** Strains used in this study. **Table S2.** Primers used in this study. **Table S3.** Gene IDs and protein functions of promoters.

**Additional file 2: Table S4. **1. Comparative genomic analysis of WT VS STA. 2. Key mutations in the evolved strain STA. 3. Differentially expressed gene of WT VS WT-3g. 4. Differentially expressed gene of STA VS STA-3g. 5. Differentially expressed gene of WT VS STA. 6. Differentially expressed gene of WT-3g VS STA-3g.

## Data Availability

All data generated or analyzed during this study are included in this published article and its additional files.
