# Supplementary material for: Efficient biosynthesis of (R)-mandelic acid from styrene oxide by an adaptive evolutionary Gluconobacter oxydans STA
Source: Biotechnol Biofuels Bioprod. 2023 Jan 13;16:8. doi: 10.1186/s13068-023-02258-7 (PMC9838050; doi:10.1186/s13068-023-02258-7)
Supplement: Supplementary file 1 — Additional file 1: Fig. S1 (a) SDS–PAGE of SpEH expression strains. Lane M: protein marker; Lane 1: whole cell protein of wild-type G. oxydans without overexpressing SpEH; Lane 2: whole cell protein of WT-SpEH; Lane 3: whole cell protein of G. oxydans STA without overexpressing SpEH; Lane 4: whole cell protein of STA-SpEH. (b) Enzyme activity of crude SpEH of WT-SpEH and STA-SpEH. Fig. S2. Membrane permeability and hydrophobicity analysis of G. oxydans STA strains. (a) NPN fluorescence intensity analyses of outer membrane permeability in STA stains. (b) Inner membrane permeability change of propidium iodide (PI) uptake factor in STA stains. (c) Membrane hydrophobicity was changed STA stains. **P < 0.01. ***P < 0.001. Table S1. Strains used in this study. Table S2. Primers used in this study. Table S3. Gene IDs and protein functions of promoters. [file 13068_2023_2258_MOESM1_ESM.docx]

Additional Materials

**Efficient biosynthesis of (*R*)-mandelic acid from styrene oxide by an adaptive evolutionary *Gluconobacter oxydans* STA**

Fei Liu^1^, Junping Zhou^2^, Mengkai Hu^1^, Yan Chen^1^, Jin Han ^1^, Xuewei Pan^1^, Jiajia You^1^, Meijuan Xu^1^, Taowei Yang^1^, Minglong Shao^1^, Xian Zhang^1^* and Zhiming Rao^1^*

1 Key Laboratory of Industrial Biotechnology of the Ministry of Education, Laboratory of Applied Microorganisms and Metabolic Engineering, School of Biotechnology, Jiangnan University, Wuxi 214122, China

2 School of Biotechnology, Zhejiang University of Technology, Hangzhou 310014, China.

* Correspondence to: Xian Zhang, E-mail: zx@jiangnan.edu.cn; Zhiming Rao, E-mail: raozhm@jiangnan.edu.cn.


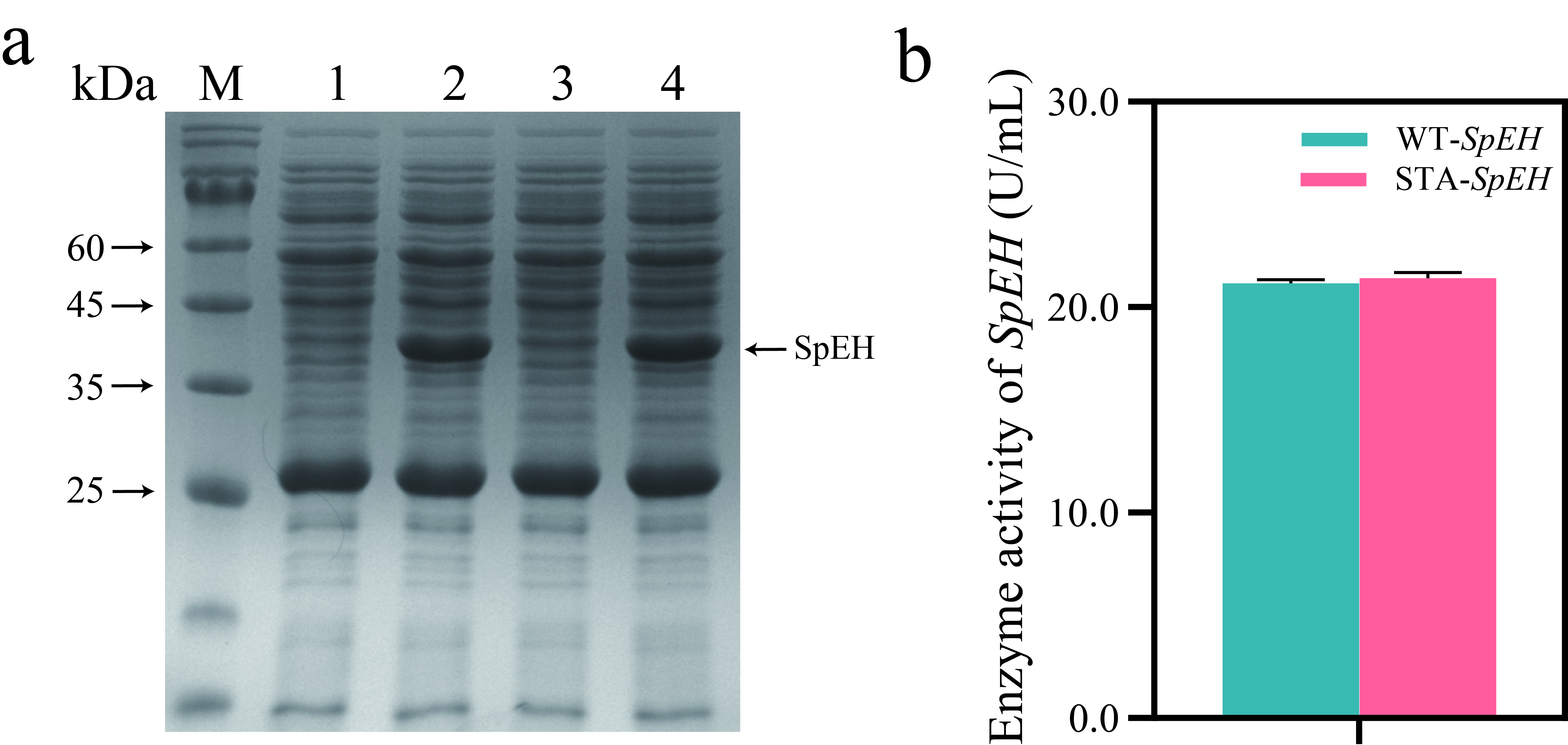


**Fig. S1** (a) SDS-PAGE of *SpEH* expression strains. Lane M: protein marker; Lane 1: whole cell protein of wild-type *G. oxydans* without overexpressing SpEH; Lane 2: whole cell protein of WT-*SpEH*; Lane 3: whole cell protein of *G. oxydans* STA without overexpressing SpEH; Lane 4: whole cell protein of STA-*SpEH*. (b) Enzyme activity of crude SpEH of WT-*SpEH* and STA-*SpEH*.


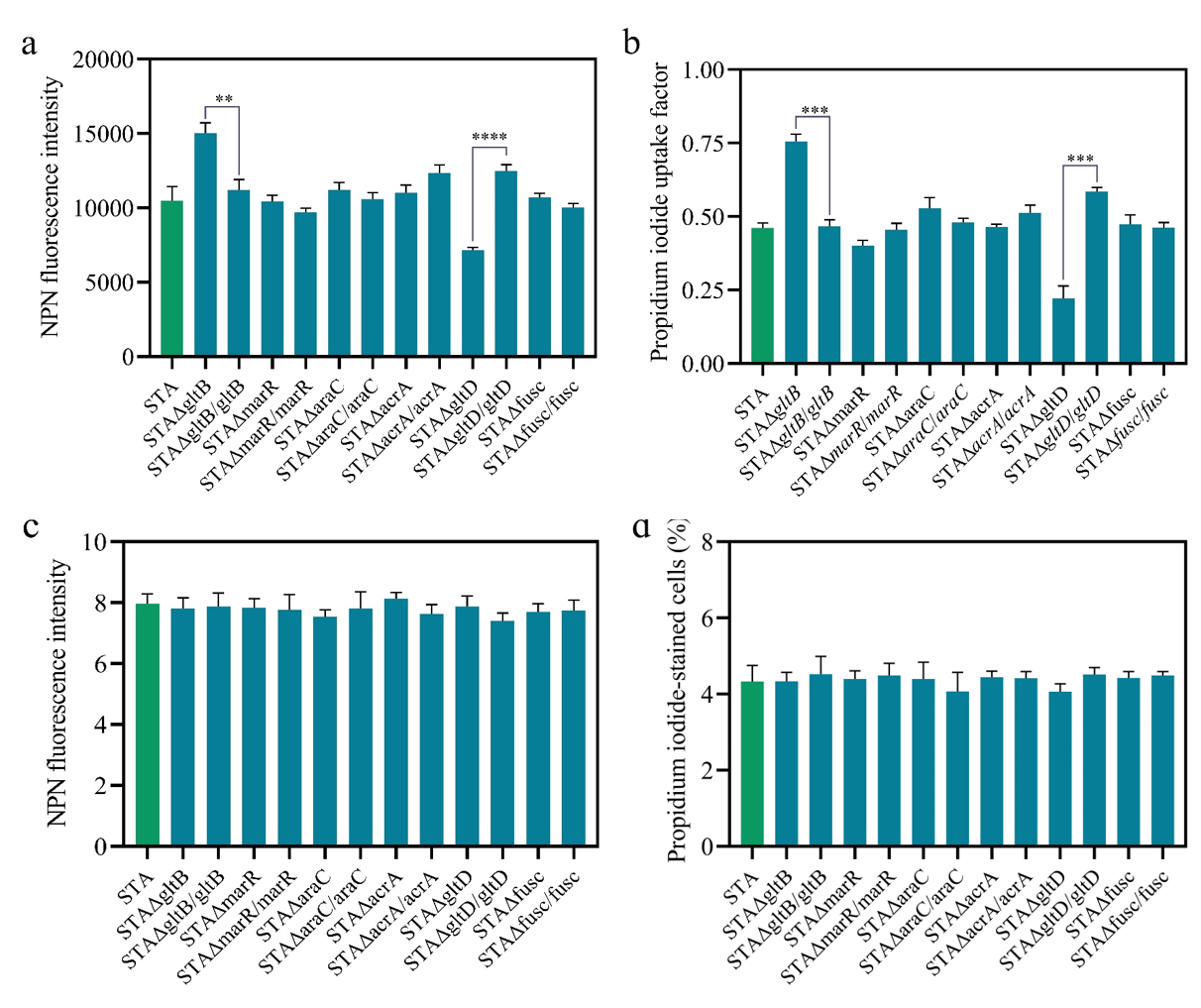


**Fig. S2** Membrane permeability and hydrophobicity analysis of *G. oxydans* STA strains. (a) NPN fluorescence intensity analyses of outer membrane permeability in STA stains. (b) Inner membrane permeability change of propidium iodide (PI) uptake factor in STA stains. (c) Membrane hydrophobicity was changed STA stains. **P < 0.01. ***P < 0.001.

**Table S1:** Strains used in this study

| Strains/Plasmids | Characteristics | Source |
| --- | --- | --- |
| **strains** |  |  |
| *G. oxydans* 621H | The expression host | This lab |
| *G. oxydans* STA | *G. oxydans* 621H derivate, tolerates high concentrations of R-MA | This study |
| *E. coli* JM109 | The cloning host | This lab |
| WT-P*_12780_*-*eGFP* | *G. oxydans* 621H derivate, expressing *eGFP* under the control of P*_12780_* via the plasmid pBBR1MCS-2 | This study |
| WT-P*_04000_*- *eGFP* | *G. oxydans* 621H derivate, expressing *eGFP* under the control of P*_04000_* via the plasmid pBBR1MCS-2 | This study |
| WT-P*_04650_*- *eGFP* | *G. oxydans* 621H derivate, expressing *eGFP* under the control of P*_04650_* via the plasmid pBBR1MCS-2 | This study |
| WT-P*_04750_*- *eGFP* | *G. oxydans* 621H derivate, expressing *eGFP* under the control of P*_04750_* via the plasmid pBBR1MCS-2 | This study |
| WT-P*_10190_*- *eGFP* | *G. oxydans* 621H derivate, expressing *eGFP* under the control of P*_10190_* via the plasmid pBBR1MCS-2 | This study |
| WT-P*_09400_*- *eGFP* | *G. oxydans* 621H derivate, expressing *eGFP* under the control of P*_09400_* via the plasmid pBBR1MCS-2 | This study |
| WT-P*_02805_*- *eGFP* | *G. oxydans* 621H derivate, expressing *eGFP* under the control of P*_02805_* via the plasmid pBBR1MCS-2 | This study |
| WT-P*_14120_*- *eGFP* | *G. oxydans* 621H derivate, expressing *eGFP* under the control of P*_14120_* via the plasmid pBBR1MCS-2 | This study |
| WT-P*_05500_*- *eGFP* | *G. oxydans* 621H derivate, expressing *eGFP* under the control of P*_05500_* via the plasmid pBBR1MCS-2 | This study |
| WT-P*_10775_*- *eGFP* | *G. oxydans* 621H derivate, expressing *eGFP* under the control of P*_10775_* via the plasmid pBBR1MCS-2 | This study |
| WT-P*_tufB_*- *eGFP* | *G. oxydans* 621H derivate, expressing *eGFP* under the control of P*_tufB_* via the plasmid pBBR1MCS-2 | This study |
| WT-P*_dnak_*- *eGFP* | *G. oxydans* 621H derivate, expressing *eGFP* under the control of P*_dnak_* via the plasmid pBBR1MCS-2 | This study |
| WT-P*_lac_*- *eGFP* | *G. oxydans* 621H derivate, expressing *eGFP* under the control of the original promoter P*_lac_* of the plasmid pBBR1MCS-2 | This study |
| WT-P*_12780_*-*SpEH*  (WT-*SpEH*) | *G. oxydans* 621H derivate, expressing *SpEH* under the control of P*_12780_* via the plasmid pBBR1MCS-2 | This study |
| WT-P*_04000_*-*SpEH* | *G. oxydans* 621H derivate, expressing *SpEH* under the control of P*_04000_* via the plasmid pBBR1MCS-2 | This study |
| WT-P*_04650_*-*SpEH* | *G. oxydans* 621H derivate, expressing *SpEH* under the control of P*_04650_* via the plasmid pBBR1MCS-2 | This study |
| WT-P*_04750_*-*SpEH* | *G. oxydans* 621H derivate, expressing *SpEH* under the control of P*_04750_* via the plasmid pBBR1MCS-2 | This study |
| WT-P*_10190_*-*SpEH* | *G. oxydans* 621H derivate, expressing *SpEH* under the control of P*_10190_* via the plasmid pBBR1MCS-2 | This study |
| WT-P*_09400_*-*SpEH* | *G. oxydans* 621H derivate, expressing *SpEH* under the control of P*_09400_* via the plasmid pBBR1MCS-2 | This study |
| WT-P*_02805_*-*SpEH* | *G. oxydans* 621H derivate, expressing *SpEH* under the control of P*_02805_* via the plasmid pBBR1MCS-2 | This study |
| WT-P*_tufB_*-*SpEH* | *G. oxydans* 621H derivate, expressing *SpEH* under the control of P*_tufB_* via the plasmid pBBR1MCS-2 | This study |
| WT-P*_dnak_*-*SpEH* | *G. oxydans* 621H derivate, expressing *SpEH* under the control of P*_dnak_* via the plasmid pBBR1MCS-2 | This study |
| WT-P*_lac_*-*SpEH* | *G. oxydans* 621H derivate, expressing *SpEH* under the control of the original promoter P*_lac_* of the plasmid pBBR1MCS-2 | This study |
| STA- P*_12780_*-*SpEH*  (STA-*SpEH*) | *G. oxydans* STA derivate, expressing *SpEH* under the control of P*_12780_* via the plasmid pBBR1MCS-2 | This study |
| STAΔ*AraC* | *G. oxydans* STA derivate, *G. oxydans* STAΔ*araC* | This study |
| STAΔ*gltB* | *G. oxydans* STA derivate, *G. oxydans* STAΔ*gltB* | This study |
| STAΔ*gltD* | *G. oxydans* STA derivate, *G. oxydans* STAΔ*gltD* | This study |
| STAΔ*FUSC* | *G. oxydans* STA derivate, *G. oxydans* STAΔ*FUSC* | This study |
| STAΔ*MarR* | *G. oxydans* STA derivate, *G. oxydans* STAΔ*MarR* | This study |
| STAΔ*acrA* | *G. oxydans* STA derivate, *G. oxydans* STAΔ*acrA* | This study |
| STAΔ*AraC/AraC* | *G. oxydans* STAΔ*araC* derivate, expressing *araC* via pBB-5 | This study |
| STAΔ*gltB/gltB* | *G. oxydans* STAΔ*gltB* derivate, expressing *gltB* via pBB-5 | This study |
| STAΔ*gltD/gltD* | *G. oxydans* STAΔ*gltD* derivate, expressing *gltD* via pBB-5 | This study |
| STAΔ*FUSC/FUSC* | *G. oxydans* STAΔ*FUSC* derivate, expressing *FUSC* via pBB-5 | This study |
| STAΔ*MarR/MarR* | *G. oxydans* STAΔ*MarR* derivate, expressing *MarR* via pBB-5 | This study |
| STAΔ*acrA/acrA* | *G. oxydans* STAΔacrA derivate, expressing *acrA* via pBB-5 | This study |
| WT-*acrA* | *G. oxydans* 621H derivate, expressing *acrA* via pBB-5 | This study |
| STA-*acrA* | *G. oxydans* STA derivate, expressing *acrA* via pBB-5 | This study |
| WT-*SpEH/acrA* | *G. oxydans* 621H derivate, coexpressing *SpEH* and *acrA* via pBB-5; gene *SpEH* was controled by P*_12780_* and gene *acrA* by P*_lac_* | This study |
| STA-*SpEH/acrA* | *G. oxydans* STA derivate, coexpressing *SpEH* and *acrA* via pBB-5; gene *SpEH* was controled by P*_12780_* and gene *acrA* by P*_lac_* | This study |
| **plasmids** |  | This study |
| pBB-2 | Kan^r^, *E. coli*-*G. oxydans* shuttle vector pBBR1MCS-2 | This lab |
| pBB-5 | Gm^r^, *E. coli*-*G. oxydans* shuttle vector pBBR1MCS-5 | This lab |

**Table S2:** Primers used in this study

| Primers | Sequence (5'-3') |
| --- | --- |
| PBB-*HindIII*-*GFP*-R | caggaattcgatatcaagcttTTATTTGTATAGTTCATCCATG |
| *GFP*-P*14120*-F | CCGGAGCAGTGTCTGCGCCGATGGGTAAGGGAGAAGAAC |
| P*14120*-*GFP*-R | GTTCTTCTCCCTTACCCATCGGCGCAGACACTGCTCCGG |
| pbb-promoter-P*14120*-F | gtaccgggccccccctcgagGAAAGCGGTGCTCGGGTGCG |
| *GFP*-P*02805*-F | CAAGACGGAAGGATCCGGTCATGGGTAAGGGAGAAGAAC |
| P*02805*-GFP-R | GTTCTTCTCCCTTACCCATGACCGGATCCTTCCGTCTTG |
| pbb-P*02805*-prompter-F | gtaccgggccccccctcgagGTAGGCGGCGGACCATCGGG |
| *GFP*-P*10775*-F | GCCATAAATGGAGCGATCCATGGGTAAGGGAGAAGAAC |
| P*10775*-*GFP*-R | GTTCTTCTCCCTTACCCATGGATCGCTCCATTTATGGC |
| pbb-P*10775*-promoter-F | gtaccgggccccccctcgagCGACTCCGCGGAGCCGGCTTC |
| *GFP*-P*04650*--F | CGATGTTCAGGAGTAGTCCAATGGGTAAGGGAGAAGAAC |
| P*04650*-*GFP*-R | GTTCTTCTCCCTTACCCATTGGACTACTCCTGAACATCG |
| PBB-P*04650*-promoter-F | gtaccgggccccccctcgagAGAAGCGCCGAAGCGGCGAC |
| *GFP*-P*dnak*-F | CGCTGAGAGGACTGATATTACATGGGTAAGGGAGAAGAAC |
| P*dnak*-*GFP*-R | GTTCTTCTCCCTTACCCATGTAATATCAGTCCTCTCAGCG |
| PBB-P*dnak*-promoter-F | gtaccgggccccccctcgagGCGGGAGTATTCGAGCTGGTC |
| *GFP*-P*05500*-F | GGTTCCACAGTAGAGGGCACTATGGGTAAGGGAGAAGAAC |
| P*05500*-*GFP*-R | GTTCTTCTCCCTTACCCATAGTGCCCTCTACTGTGGAACC |
| PBB-P*05500*-promoter-F | gggtaccgggccccccctcgagCGTCAGCAACAACGTAAATG |
| *GFP*-P*04000*-F | GAATATGATCCGTTATTTCATGGGTAAGGGAGAAGAAC |
| P*04000*-*GFP*-R | GTTCTTCTCCCTTACCCATGAAATAACGGATCATATTC |
| PBB-P*04000*-Promoter-F | gtaccgggccccccctcgagGCCGCGAACTGGCGGAATGG |
| *GFP*-P*12780*-F | GTGACATGAAAAGGATTACGAAATGGGTAAGGGAGAAGAAC |
| P*12780*-*GFP*-R | GTTCTTCTCCCTTACCCATTTCGTAATCCTTTTCATGTCAC |
| PBB-P*12780*-promoter-F | gtaccgggccccccctcgagTGGAGAAATATCCGCAGTTTC |
| *GFP*-P*09400*-F | CTGAGATGAAGGAGCCTGACAATGGGTAAGGGAGAAGAAC |
| P*09400*-*GFP*-R | GTTCTTCTCCCTTACCCATTGTCAGGCTCCTTCATCTCAG |
| PBB-P*09400*-Promoter-F | gtaccgggccccccctcgagGGCGGTACGCCGTCCTGGGG |
| *GFP*-P*10190*-F | GTCACTAAGAGGACGAAAACATGGGTAAGGGAGAAGAAC |
| P*10190*-*GFP*-R | GTTCTTCTCCCTTACCCATGTTTTCGTCCTCTTAGTGAC |
| PBB-P*10190*-promoter-F | gtaccgggccccccctcgagGACTGGTTCCGGGAGTCGTTC |
| *GFP*-P*tufB*-F | CGGGGTTTTGGAGAAAGACGATGGGTAAGGGAGAAGAAC |
| P*tufB*-*GFP*-R | GTTCTTCTCCCTTACCCATCGTCTTTCTCCAAAACCCCG |
| PBB-P*tufB*-Promoter-F | gtaccgggccccccctcgagGGAATGAGTCGCCGTCACCG |
| PBB-GFP-F | ggtaccgggccccccctcgagaATGGGTAAGGGAGAAGAAC |
| *GFP*-P*04750*-F | CTGTATATGGAAAGAAGAGTGCCATGGGTAAGGGAGAAGAAC |
| P*04750*-*GFP*-R | GTTCTTCTCCCTTACCCATGGCACTCTTCTTTCCATATACAG |
| *SpEH*-P*02905*-F | CAAGACGGAAGGATCCGGTCATGAACGTTGAACACATCC |
| P*02805*-*SpEH*-R | GGATGTGTTCAACGTTCATGACCGGATCCTTCCGTCTTG |
| *SpEH*-P*04650*-F | CGATGTTCAGGAGTAGTCCAATGAACGTTGAACACATCC |
| P*04650*-*SpEH*-R | GGATGTGTTCAACGTTCATTGGACTACTCCTGAACATCG |
| *SpEH*-P*04000*-F | GAATATGATCCGTTATTTCATGAACGTTGAACACATCC |
| P*04000*-*SpEH*-R | GGATGTGTTCAACGTTCATGAAATAACGGATCATATTC |
| *SpEH*-P*09400*-F | CTGAGATGAAGGAGCCTGACAATGAACGTTGAACACATCC |
| P*09400*-*SpEH*-R | GGATGTGTTCAACGTTCATTGTCAGGCTCCTTCATCTCAG |
| *SpEH*-P*10190*-F | GTCACTAAGAGGACGAAAACATGAACGTTGAACACATCC |
| P*10190*-*SpEH*-R | GGATGTGTTCAACGTTCATGTTTTCGTCCTCTTAGTGAC |
| *SpEH*-P*04750*-F | GTATATGGAAAGAAGAGTGCCATGAACGTTGAACACATCC |
| P*04750*-*SpEH*-R | GGATGTGTTCAACGTTCATGGCACTCTTCTTTCCATATAC |
| *SpEH*-P*dnak*-F | GCTGAGAGGACTGATATTACATGAACGTTGAACACATCCG |
| P*dnak*-*SpEH*-R | GGATGTGTTCAACGTTCATGTAATATCAGTCCTCTCAGCG |
| *SpEH*-P*tufB*-F | CGGGGTTTTGGAGAAAGACGATGAACGTTGAACACATCCG |
| P*tufB*-*SpEH*-R | CGGATGTGTTCAACGTTCATCGTCTTTCTCCAAAACCCCGC |
| PBB-*gltB*-F | ccgggccccccctcgaggATGACACAGAATAACGATTTC |
| pbb-*gltB*-R | caggaattcgatatcaagcttTCAGGCCGAAAGCTGGG |
| PBB-*gltD*-F | gtaccgggccccccctcgaggATGGCCGAACGCATGTTGC |
| PBB-*gltD*-R | ggaattcgatatcaagcttTCATTCCGCGGCCTCCAGAG |
| PBB-*FUSC*-F | gtaccgggccccccctcgaggATGCGCAATCCTTTTCGCC |
| PBB-*FUSC*-R | ggaattcgatatcaagcttTCACCGCGCAGGATTGACCG |
| PBB-*MarR*-F | gtaccgggccccccctcgagaATGGCTGTTCAGTACGATCC |
| PBB-*MarR*-R | caggaattcgatatcaagcttTCAGGGGGAAGAAAAACCG |
| pbb-2-*acrA*-F | gtaccgggccccccctcgagaGTGAGCCATTCTGCGGATCAG |
| PBB-2-*acrA*-R | caggaattcgatatcaagcttTCAGCGAGATCCGGCCTC |
| PBB-2-*AraC*-F | gtaccgggccccccctcgagaATGAGCACAGCTACGACAATTTTAG |
| PBB-2-*AraC*-R | caggaattcgatatcaagcttCTAATCCTGCACTCCTAAATG |
| *FUSC*-up-F | GATGGCCAGGGCCAGGACGATGAG |
| *FUSC*-up-*kan*-R | cttcttgacgagttcttctgaGAAAGGTCCGGAAAATCAGG |
| *kan*-*FUSC*-up-F | CTGATTTTCCGGACCTTTCtcagaagaactcgtcaagaaggc |
| *kan*-*FUSC*-down-R | GAATGTCGCGGACATTATCGACaccttcgggagcgcctgaag |
| *FUSC*-down-*kan*-F | cttcaggcgctcccgaaggtGTCGATAATGTCCGCGACATTC |
| *FUSC*-down-R | CACGACACCAGACGGACACGGTAC |
| *gltB*-up-F | GGCGCAGTGTCGGGATCGTGGGATC |
| *gltB*-up-*kan*-R | cttcttgacgagttcttctgaTCCGCGGCCTCCAGAGAACC |
| *kan*-*gltB*-up-F | GTTCTCTGGAGGCCGCGGAtcagaagaactcgtcaagaag |
| *kan*-*gltB*-down-R | CTTCCTTCAGATCCAGCGCCaccttcgggagcgcctgaag |
| *gltB*-down-*kan*-F | cttcaggcgctcccgaaggtGGCGCTGGATCTGAAGGAAGG |
| *gltB*-down-R | CCAGCGCGCGGGACAGCCCGATC |
| *gltD*-up-F | GAAGCTCGAGGCGGAAATTCTGG |
| *gltD*-up-*kan*-R | cttcttgacgagttcttctgaGCGTCGTGTCTCCTGCAAAAC |
| *kan*-*gltD*-up-F | GTTTTGCAGGAGACACGACGCtcagaagaactcgtcaagaaggc |
| *kan*-*gltD*-down-R | GATGAAATCGTTATTCTGTGaccttcgggagcgcctgaagcc |
| *gltD*-down-*kan*-F | cttcaggcgctcccgaaggtCACAGAATAACGATTTCATCC |
| *gltD*-down-R | GCACCGCCAGAGGCCGGCACCAGC |
| *acrA*-up-F | GCCGAGGCAGCCATCTGCCGCTCGGCAATG |
| *acrA*-up-*kan*-R | cttcttgacgagttcttctgaTGAAACCGGAATGTTCCCAG |
| *kan*-*acrA*-up-F | GACTGGGAACATTCCGGTTTCAtcagaagaactcgtcaagaagg |
| *kan*-*acrA*-down-R | CTGGGCCTGAAGGAGACCGCGCgagatgcgcaagaaggccgatc |
| *acrA*-down-*kan*-F | cggccttcttgcgcatctcGCGCGGTCTCCTTCAGGCCCAG |
| *acrA*-down-R | CTCCATCCAGGAGCCGATGACGCAGCCGAC |
| *AraC*-up-F | GGGTTTGATGGTGTGATATTTTCACGAGAG |
| *AraC*-up-*kan*-R | cttgacgagttcttctgaGTGTTTGGTCCCGGGGTTTG |
| *kan*-*AraC*-up-F | CAAACCCCGGGACCAAACACtcagaagaactcgtcaag |
| *kan*-*AraC*-down-R | CCTGATACCATTCTCGCCgagatgcgcaagaaggccg |
| *AraC*-down-*kan*-F | cggccttcttgcgcatctcGGCGAGAATGGTATCAGG |
| *AraC*-down-R | CCTAGACTAGCGCGACATCGCTTACGCG |
| *MarR*-up-F | GTGTGCATCGATCACCGCGAG |
| *MarR*-up-*kan*-R | cttcttgacgagttcttctgaTTCTGCTGACACCTCATCTG |
| *kan*-*MarR*-up-F | GATGAGGTGTCAGCAGAAtcagaagaactcgtcaagaag |
| *kan*-*MarR*-down-R | GCATGAAAGGTCCGGAAAAgagatgcgcaagaaggccg |
| *MarR*-down-*kan*-F | cggccttcttgcgcatctcTTTTCCGGACCTTTCATGC |
| *MarR*-down-R | CTTCCAGACAGGCCAGCACG |
| P*12780*-*acrA*-F | GAGGCCGGATCTCGCTGATGGAGAAATATCCGCAGTTTCTTC |
| *acrA*-P*12780*-R | GAAGAAACTGCGGATATTTCTCCATCAGCGAGATCCGGCCTC |

**Table S3:** Gene IDs and protein functions of promoters

| Promoters | Gene ID | Functions |
| --- | --- | --- |
| P*_14120_* | GOX_RS14120 | RNase P RNA component class A |
| P*_02805_* | GOX_RS02805 | CsbD family protein |
| P*_10775_* | GOX_RS10775 | co-chaperone GroES |
| P*_04650_* | GOX_RS04650 | DNA starvation/stationary phase protection protein Dps |
| P*_04750_* | GOX_RS04750 | DUF2501 domain-containing protein |
| P*_05500_* | GOX_RS05500 | ferrochelatase |
| P*_04000_* | GOX_RS04000 | hypothetical protein |
| P*_10190_* | GOX_RS10190 | OmpA family protein |
| P*_12780_* | GOX_RS12780 | hypothetical protein |
| P*_09400_* | GOX_RS09400 | YidB family protein |
| P*_dnaK_* | GOX_RS05415 | molecular chaperone DnaK |
| P*_tufB_* | GOX_RS03065 | elongation factor Tu |
